# Prevention of abdominal wound infection (PROUD trial, DRKS00000390): study protocol for a randomized controlled trial

**DOI:** 10.1186/1745-6215-12-245

**Published:** 2011-11-21

**Authors:** Ulrike Heger, Sabine Voss, Phillip Knebel, Colette Doerr-Harim, Jens Neudecker, Christoph Schuhmacher, Eugen Faist, Markus K Diener, Meinhard Kieser, Christoph M Seiler, Markus W Büchler

**Affiliations:** 1 Study Centre of the German Surgical Society, Department of General, Visceral and Transplantation Surgery, University of Heidelberg, Im Neuenheimer Feld 110, 69120 Heidelberg, Germany; 2 Department of General, Visceral, Vascular, and Thoracic Surgery, Campus Charité Mitte, Charitéplatz 1, 10117 Berlin, Germany; 3 Department of Surgery, Klinikum rechts der Isar, Technische Universität München, Ismaningerstr. 22, 81675 München, Germany; 4 Department of Surgery, Klinikum Grosshadern, Ludwig Maximilians University Munich, Marchioninistraße 15, 81377 München, Germany; 5 Institute of Medical Biometry and Informatics, University of Heidelberg, Im Neuenheimer Feld 305, 69120 Heidelberg, Germany

## Abstract

**Background:**

Wound infection affects a considerable portion of patients after abdominal operations, increasing health care costs and postoperative morbidity and affecting quality of life. Antibacterial coating has been suggested as an effective measure to decrease postoperative wound infections after laparotomies. The INLINE metaanalysis has recently shown the superiority of a slowly absorbable continuous suture for abdominal closure; with PDS plus^® ^such a suture has now been made available with triclosan antibacterial coating.

**Methods/Design:**

The PROUD trial is designed as a randomised, controlled, observer, surgeon and patient blinded multicenter superiority trial with two parallel groups and a primary endpoint of wound infection during 30 days after surgery. The intervention group will receive triclosan coated polydioxanone sutures, whereas the control group will receive the standard polydioxanone sutures; abdominal closure will otherwise be standardized in both groups. Statistical analysis is based on intention-to-treat population via binary logistic regression analysis, the total sample size of n = 750 is sufficient to ensure alpha = 5% and power = 80%, an interim analysis will be carried out after data of 375 patients are available.

**Discussion:**

The PROUD trial will yield robust data to determine the effectiveness of antibacterial coating in one of the standard sutures for abdominal closure and potentially lead to amendment of current guidelines. The exploration of clinically objective parameters as well as quality of life holds immediate relevance for clinical management and the pragmatic trial design ensures high external validity.

**Trial Registration:**

The trial protocol has been registered with the German Clinical Trials Register (DRKS00000390).

## Background

### Rationale

Postoperative surgical site infections (SSI) are one of the most common complications after laparotomy. Many strategies have been developed to reduce this burden such as the introduction of less invasive procedures (e.g. laparoscopic interventions) but still most of the intraabdominal procedures are performed as open surgeries worldwide. Preoperative antibiotic prophylaxis besides routine use of effective and persistent skin antisepsis as well as avoidance and/or control of contamination were amongst the most effective interventions introduced in the last century to reduce SSI.

Currently about 12% of patients undergoing elective open colorectal procedures develop an SSI [1]. This is in accordance with the "Hospital in Europe Link for Infection Control through Surveillance" (HELICS) SSI statistical report that has reported a similar incidence of SSI [2]. The INSECT multicenter RCT focussing on different strategies for abdominal fascia closure after elective primary midline laparotomy in various surgical indications detected a wound infection rate of 16% as a secondary outcome [3]. Therefore further efforts are necessary to reduce this problem which may cause impairment of the patients' quality of life, require additional wound therapy treatment, lead to prolongation of hospital stay or delay of further relevant treatments (e.g. start of adjuvant chemotherapy) and increases the risk for further complications such as wound dehiscence or burst abdomen requiring additional surgery [4]. Today 75% of all SSI are superficial incisional infections whereas the remaining 25% are deep incisional or deep organ space SSI.

Patient-related factors such as comorbidities (e.g. diabetes mellitus) or life style habits (e.g. smoking) have to be taken into account but are difficult to change once an intervention is needed. Therefore further efforts on the surgeon's side are required to reduce the frequency of SSI. Any foreign material such as sutures needed for closure of the abdominal fascia increases the risk of a SSI, and it has been shown that bacteria not only colonize wound tissues but the actual suture itself. Therefore various suture materials have been investigated for several decades to improve wound healing and reduce infection rates [5].

### Preliminary data

Triclosan is an antiseptic known to interfere with microbial lipidsynthesis [6] and has been used to cover suture material in order to reduce SSI. Two recent historically controlled studies comparing the interrupted closure of the abdominal fascia with a triclosan-coated braided rapidly absorbable suture material (polyglactin 910, Vicryl plus^®^) versus a slowly absorbable continuous loop suture (polydioxanone, PDS II^®^) in patients with elective or emergency surgery demonstrated a highly significant reduction of wound infections using the coated material [7,8]. This is in accordance with data from in vitro studies showing a considerable decrease in bacterial adherence with triclosan-coated sutures [9]. Two more studies demonstrated antibacterial efficacy of the triclosan-coating in Vicryl sutures in experimental large animal studies [10,11]. So far, however, only one multicenter randomized controlled trial comparing the non-coated with the coated PDS suture has been conducted but not yet published (Baracs J, Huszár O, Horváth Ö: Abdominal Wall Closure With Triclosan-coated Suture; *NCT01123616*.). After elective midline incisions the abdominal fascia should be closed with a slowly absorbable continuous suture for best prevention of incisional hernias according to the results of a recent systematic review and meta-analysis [12]. Whether coating of the sutures with antibacterial substances is beneficial in clinical practice has yet to be determined.

SSI is not a problem of open colorectal surgery alone. The study populations of the INSECT Trial and the historically controlled studies by Justinger et al are covering a much larger spectrum of patients and therefore a reduction of SSI should be demonstrable in all these patients [3,7,8] Thus, the PROUD trial was planned as a multicenter effectiveness RCT to investigate if SSI can be clinically relevant reduced with a triclosan-coated PDS suture.

### Objective and hypotheses

The objective of the PROUD trial is to yield reliable data on the effectiveness of triclosan-coated PDS suture for abdominal facia closure in preventing SSI compared to non-coated PDS sutures. The null hypothesis to be tested in confirmatory analysis states that the rate of superficial and deep incisional SSI within 30 days after midline incision is equal in both treatment groups.

## Methods/Design

### Trial locations

The trial locations comprise 22 surgical departments of secondary and tertiary care across Germany (Table 1).

**Table 1:** Trial Locations (Germany)

Institutions	Principal Investigators
Department of General and Visceral Surgery, Campus Mitte, Charite-Universitaetsmedizin Berlin, Berlin	Prof. Dr. Joachim M. Müller PD Dr. Jens Neudecker

Department of General, Visceral and Transplantation Surgery, Virchow Klinikum, Charite-Universitaetsmedizin Berlin, Berlin	PD Dr. Daniel Seehofer

Gemeinschaftskrankenhaus Havelhöhe, Medizinische Klinik Schwerpunkt Chirurgie, Berlin	PD Dr. Hans-Peter Lemmens

Department of Surgery, Vivantes Klinikum Neukölln, Berlin	Prof. Dr. Bartholomäus Böhm

Department of General, Visceral and Minimally Invasive Surgery, Park-Klinik Weissensee, Berlin	PD Dr. Georg Arlt

Department of General and Visceral Surgery, Sana Klinikum Lichtenberg, Berlin	Prof. Dr. Klaus Gellert

Department of General and Visceral Surgery, St. Josef Krankenhaus Berlin Tempelhof, Berlin	Prof. Dr. Reiner Kunz

Department of General and Visceral Surgery, Unfallkrankenhaus Berlin, Berlin	Dr. Henryk Thielemann

Department of General, Visceral and Trauma Surgery, St.-Josefs-Hospital Dortmund-Hörde, Dortmund	Dr. Erwin Stein

Division of General and Visceral Surgery, University Medical Center Freiburg, Freiburg	Prof. Dr. Oliver Thomusch

Department for General and Visceral Surgery, Universitaetsmedizin Goettingen, Goettingen	Prof. Dr. Heinz Becker

Department for General and Visceral Surgery, Asklepios Klinik Harburg, Hamburg	Prof. Dr. Friedrich Kallinowski

Department of Surgery, Krankenhaus Salem, Heidelberg	PD Dr. Moritz v. Frankenberg

Department of General, Visceral and Transplantation Surgery, University of Heidelberg, Heidelberg	Dr. Markus Diener

Department of General, Visceral and Trauma Surgery, Krankenhaus der Augustinerinnen, Koeln	Prof. Dr. K. Tobias E. Beckurts

Department of Surgery, University Medical Center Schleswig-Holstein, Campus Luebeck, Luebeck	PD Dr. Dr. Uwe J. Roblick

Department of General and Abdominal Surgery, Johannes Gutenberg-University Hospital, Mainz.	Dr. Boris Jansen-Winkeln

Department of Surgery, University Hospital Campus Grosshadern, Muenchen	Prof. Dr. Karl-Walter Jauch

Department of Surgery, Klinikum Neumarkt, Neumarkt	Dr. Manfred Kästel

Department of Surgery, Visceral, Thoracic and Vascular Surgery, Ernst Moritz Arndt University, Greifswald, Germany	Prof. Dr. med. Claus-Dieter Heidecke

Department of General, Visceral and Thoracic Surgery, Klinikum am Steinenberg Reutlingen, Reutlingen	Prof. Dr. Thomas Zimmermann Dr. René Hodina

Department of General, Visceral and Thoracic Surgery, Marienhospital, Stuttgart	Dr. Julius Pochhammer

### Trial population and eligibility criteria

Patients who will undergo elective and midline abdominal laparotomy for any reason will be recruited for this trial. All patients will be informed about the purpose of the trial, the operation modalities, and their benefits as well as risks. Patients will be asked whether they are prepared to participate in the trial prior to their inclusion. After being screened for the inclusion and exclusion criteria eligible patients will be included into the trial. Patients who were screened but not enrolled in the trial (including patients unable to give informed consent due to any reason) will be documented in the screening log, recording the reason for exclusion.

#### Inclusion criteria

• Age equal or greater than 18 years

• Patients undergoing elective midline laparotomy for any reason

• Informed consent

#### Exclusion criteria

• Participation in another intervention-trial with interference of intervention and/or outcome of this study

• Impaired mental state or language problems

### Sample size

375 patients per group (including the expected drop outs) will be randomized for this trial, accounting for a total of 750 patients. This number of patients may be changed based on the results of an adaptive interim analysis, which will be performed after availability of results for the primary endpoint for a total of 375 randomized patients (i.e. 50% of required patients for a fixed sample size design). The maximum sample size is 1200 patients.

### Type of trial

**Figure 1:**
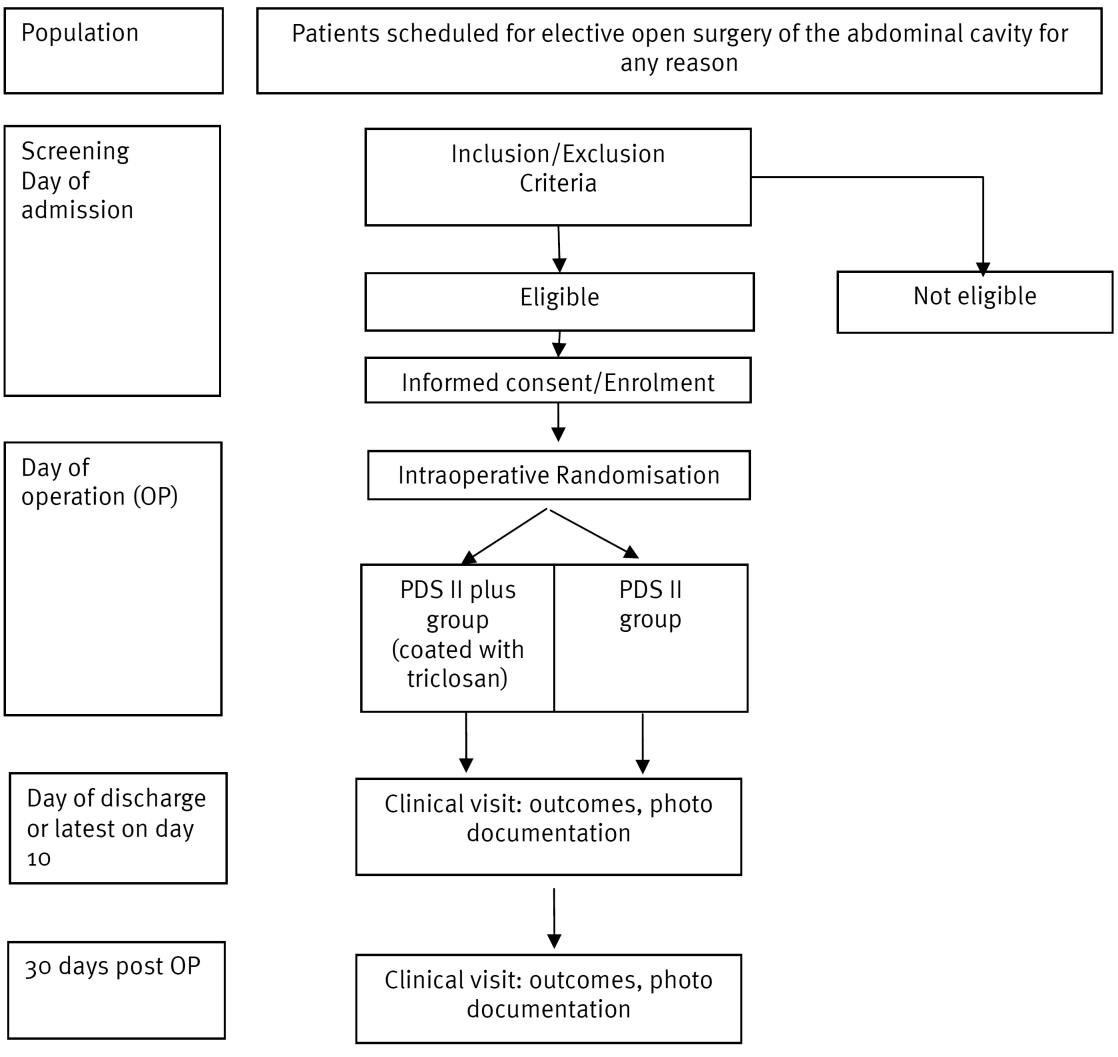
**Flow Chart PROUD Trial (see separate.jpg file)**. Timeline and course of trial participants.

Multicenter observer, surgeon and patient blinded adaptive randomized surgical trial with two parallel groups. For trial flow chart please cf. Figure 1.

### Recruitment and trial timeline

Patient recruitment started in April 2010 as a single center trial. The start date of multicenter recruitment was in January 2011. The interim analysis is expected to be performed at the latest in September 2011, after data of 375 randomized patients are available. Depending on results of the interim analysis further proceedings and timeline will be defined.

### Baseline data

If a patient has given informed consent the baseline demographic and clinical data are documented including assessement of quality of life with the EQ-5D™ instrument. Date of surgery, antibiotic prophylaxis/therapy and surgical intervention have to be reported as well as wound status (clean, clean-contaminated, contaminated) and blood loss. The surgeon performing the abdominal wall closure and his surgical expertise are recorded.

### Randomization

Patients will be randomized to one of the two treatment groups just before closure of the abdominal wall by using a web-based tool (Randomizer^© ^Software [13]).

In order to achieve comparable groups for known and unknown risk factors randomization will be performed as block randomization with a 1:1 allocation.

A sufficient number of patients will be recruited according to the sample size calculation in order to minimize random error.

### Blinding

Participating surgeons should not be influenced in their abdominal wall closure performance and have to follow the protocol in all patients. The two suture materials cannot be differentiated by color, feel or smell; furthermore, identical needles (CTX 48 mm 1/2c) are used and therefore the surgeon cannot identify whether the suture is coated or not. The suture will be handed to the surgeon in a blinded fashion by the scrub nurse to prevent unblinding by viewing the wrapper.

Patients are blinded for the type of suture they received to ensure valid assessement of quality of life; similarly, observers assessing wound status are blinded as well. Furthermore, photographs of the abdominal wound are assessed by an independent Primary Outcome Validation Committee (POVC) consisting of three board certified surgeons who independently review all photographs without knowledge of the origin.

### Interventions

Patients undergo routine scrub and site preparation according to the established standards of the participating centers. An antibiotic prophylaxis/therapy must be performed and documented according to recently updated national guidelines of the Paul-Ehrlich Gesellschaft für Chemotherapie e.V. [14]. Antibiotics must be given prior to incision of the skin.

The operation is initiated with skin incision performed with electric cautery (yellow = cutting function: working with alternating current, the tissue is heated abruptly more than 100°C, in a way that the vapor pressure ruptures the cellular membranes explosively). The subcutaneous layer is cut with electric cautery (blue = coagulation function: heating of the tissue with alternating current-effectual slowly-approximately at 100°C to vapor the intra- and extra-cellular matrix. Tissue and vessels are shrinking, hemostasis occurs). The abdominal fascia is cut in the midline with electric cautery (blue). The abdomen is opened with scissors and then the incision of the peritoneum is completed with electric cautery (blue). The surgical procedure is carried out as usual and according to local standards regarding the indication for the intervention. Documentation of wound status, surgical procedure and in case of stoma formation is mandatory in all randomized patients.

Patients are randomized to one of the two treatment groups just before closure of the abdominal wall.

Four Mikulicz clamps or equivalent clamps are placed at the edges of the abdominal fascia. In both treatment groups closure of the abdominal wall is started either from the cranial end (surgeon is on the right side of the patient) or from the caudal end (surgeon is on the left side of the patient) of the wound. The peritoneum is not closed by a separate suture.

### Trial intervention

Closure of abdominal fascia with triclosan-coated continuous polydioxanone suture (PDS * PLUS PDP9262; lot: CA6417, CLZ854 and CLM544; needle: CTX 48 mm 1/2c.).

### Control intervention

Closure of abdominal fascia with polydioxanone suture (PDS*II Z1950; lot: CB8CGLQ0, CK8DRLQ0, CL8GBHQ0, CJ8KJLQ2 and CL8GBHQ0; needle: CTX 48 mm 1/2c).

Labels of used sutures are sent to the Institute for Medical Biometry and Informatics, University of Heidelberg, to document use of suture for a given randomized patient.

The abdomen will be closed by a continuous mass closure technique using two loops of the suture. Each loop has to be stretched once by the scrub nurse before use, in order to avoid breakage of the material. The first stitch has to be anchored cranially and caudally of the incision. The peritoneum should not be stitched to prevent entrapment between the fascial edges. After having closed half of the wound, one end of the loop is cut right below the needle. After one stitch back to the opposite edge of the fascia both ends are tied with at least four counterrotating knots. The same is done with the loop from the caudal end of the wound with intersecting the other loop at the middle of the incision with an overlap of both suture lines of at least 2 cm. Both loops may not be tied together. For every patient two loops must be used, irrespective of the length of the wound.

The distance between the stitches should maximally be 1,5 cm and the distance from the edge of the fascia should be at least 1,5 cm. The subcutaneous tissue is not sutured and no subcutaneous drainage is used. The skin is closed with clips.

The expertise of the surgeon (board certified versus no certificate) as well as the number of prior abdominal wall closures at the closure will be documented.

### Permitted and not permitted medication(s)/treatment(s)

No suture material or suture technique other than described in the protocol can be used for fascia closure. Patients have to receive antibiotic prophylaxsis prior to the incision and the fascia has to be closed using the appropriate suture to which they are randomized. Any protocol violation has to be reported with a clear description in the CRF.

Postoperative care is performed according to the principles and standard of the department (artificial organ support, antibiotics, pain treatment, fluid resuscitation, nutrition). Length of intensive care unit stay and discharge date are documented.

### Risks

No additional risks are expected for participants, since both sutures are readily commercially available after fulfilling requirements for approval by several international authorities. There have been reports on several suspected cases of allergic contact dermatitis towards triclosan-coated sutures, none of which could be verified [15].

### Outcomes (primary and secondary) and assessment

The primary endpoint superficial and deep incisional surgical site infection according to Center for Disease Control (adapted from [16]) within 30 days after surgery will be assessed during two following study visits by the outcome assessors in the participating centers (Table 1). In addition a photograph is taken at each follow up visit and sent to the Study Center of the German Surgical Society for evaluation by the Primary Outcome Validation Committee (POVC). The committee members will categorize independently which of the following applies for the primary endpoint: (1) SSI present; (2) SSI not present; (3) Wound not assessable. Depending on this information patients will be classified as to whether they have met the primary endpoint or not.

Secondary endpoints include duration of surgery, length of incision (fascia), frequency of wound dehiscence (with or without evisceration), frequency of re-operation due to wound dehiscence, postoperative intensive care unit stay, postoperative hospital stay, 30 day mortality and quality of life (QoL); the latter will be assessed by the EQ-5D™ questionnaire filled in by the patient.

### Study visits

From screening to day 30 postoperatively there will be 4 study visits, assessing demographics and baseline clinical data, eligibility criteria, randomisation, surgical intervention, photo documentation of wound, quality of life and safety; please cf. table 2 for detailed information.

**Table 2:** Study Visits in the PROUD Trial

Visit	1 (Screening)	2 (OP)	3 (Discharge or latest on day 10)	4 (30 days post OP)
Demographics and baseline clinical data	X			

Eligibility criteria	X			

Randomisation, surgical intervention		X		

Clinical visit			X	X

Photo documentation of wound			X	X

Quality of life	X		X	X

Safety		X	X	X

### Data management

All protocol-required information collected during the trial is entered by the investigator, or designated representative, in a paper-based CRF. Patients fill out the quality of life questionnaires themselves. Photographs are uploaded in a centralized database by investigators. Two weeks after a patient has completed the trial all information must be sent to the Institute of Medical Biometry and Informatics Heidelberg (IMBI) for data entry. Copies remain at the participating site for further clarifications. An explanation should be given for all missing data.

In order to ensure that the database reproduces the CRFs correctly, the IMBI accomplishes a double entry of data. The completeness, validity and plausibility of data are examined by validating programs, which thereby generate queries. The sites are obliged to clarify or explain the queries. At the end of the trial the principle investigator will retain the originals of all CRFs.

The data will be managed and analyzed according to the appropriate SOPs valid in the IMBI.

### Monitoring

Participating investigators must agree to allow trial-related monitoring, including audits, ethics committee review and regulatory inspections by providing direct access to source data/documents as required. Patients' informed consent for this will also be obtained.

Monitoring will be performed using different strategies and in accordance with ICH-GCP Section 5.18. First recruitment of patients within centers will be centrally monitored by the webbased randomization tool through the Study Centre of the German Surgical Society (SDGC). If centers do not recruit a patient within a time frame of two weeks they will be contacted. If a center is not able to recruit a patient within four weeks actions will be taken including closure of sites.

Performance of follow up visits including photos will be monitored two weeks after the final visit by the IMBI. If centres do not perform according to protocol they will be notified and if more than 40% of patients randomized are not evaluable for the primary endpoint (missing photo documentation) they may be closed. On-site monitoring visits may be scheduled where there is evidence or suspicion of non-compliance by a site with important aspects of the trial requirements. Sites will be sent a letter in advance outlining the reasons for the visit, a list of documents that are to be reviewed, interviews that will be conducted, planned inspections of facilities and who will be performing the visit.

Following the monitoring visit, the monitor will provide to the site a report which will summarize the documents reviewed, along with a statement of findings, deviations, deficiencies, conclusions and actions taken or recommended.

### Safety evaluation, analysis and reporting

A serious adverse event (SAE) is any adverse event occurring at any time during the period of observation, that results in death, is immediately life-threatening, requires or prolongs hospitalization and/or results in persistent or significant disability or incapacity.

From the moment the subject has signed informed consent until the regular end of the trial at 30 days follow up or until premature withdrawal of the patient, all SAE must be documented on a "serious adverse event form" available from the investigator study file.

The following conditions are expected after the initial operation and will therefore not be classified as complication: pain, nausea, vomiting, urinary tract infection, hyper-/hypotension, imbalances of blood sugar or electrolytes and other lab values out of range, if they are not exceeding the duration and extent that can be expected after surgery.

SAE need to be reported to the SDGC once they are noticed by the investigator within a time frame of five days. The safety analysis will be based on the set of all patients for which one of the interventions was applied. SAE will be tabulated, absolute and relative frequencies will be presented; severity and relationship to the intervention will be given and compared between the groups. The Data Safety Monitoring Board will be provided an annual report of SAE during the conduction of the trial.

### Statistical methods

Superficial and deep incisional surgical site infection rates for patients in the PDS II^® ^group are estimated to occur at a rate of 0.12 [1,2]. The trials by Justinger have shown a reduction of SSI of more than 50% (from 10.8% to 4.9% and from 9.2% to 4.3% respectively). Therefore, we estimate a rate of 0.06 for PDS Plus^®^.

For a fixed sample size design, the sample size required to achieve a power of 1-β = 0.80 for the one-sided chi-square test at level α = 0.025 under these assumptions amounts to 2 × 356 = 712 (nQuery Advisor^®^, version 7.0). It can be expected that including covariates of prognostic importance in the logistic regression model as defined for the confirmatory analysis will increase the power as compared to the chi-square test. As the individual results for the primary endpoint are available within 30 days after surgery, the drop-out rate is expected to be small. Nevertheless, a potential dilution of the treatment effect due to drop-outs is taken into account (e.g. no photographs available, loss to follow up); it is assumed that this can be compensated by additional 5% of patients to be randomized, and therefore the total sample size required for a fixed sample size design amounts to n = 712 + 38 = 750 patients.

To account for the remaining uncertainty about the assumed treatment effect and the overall SSI rate, an adaptive interim analysis is performed after the results for the primary endpoint are available for a total of 375 evaluable patients. If the study is continued with a second stage, the sample size can be recalculated using the information obtained from the results of the interim analysis. The actually achieved sample size is then not fixed but random, and a variety of scenarios can be considered. If the sample size is calculated under the same assumptions with respect to the SSI rates for the two groups, applying the same the overall significance level of α = 0.025 (one-sided) but employing additionally the defined stopping boundaries and recalculating the sample size for the second stage at a conditional power of 80% on the basis of the SSI rates observed in the interim analysis results in an average total sample size of n = 766 patients; the overall power of the study is then 90% (ADDPLAN^®^, version 5.0).

### Confirmatory analysis of the primary endpoint

The null-hypothesis of equal rates of superficial and deep incisional SSI within 30 days after midline incision is tested with a logistic regression model including the covariates age, center, surgeon's experience, and BMI. An overall one-sided significance level of α = 0.025 is applied. Confirmatory analysis of the primary endpoint will be primarily based on the full analysis set which is consistent with the intention-to-treat (ITT) principle by including all patients who were randomized to one of the intervention groups. This approach reflects the idea that the study results should match as close as possible to the conditions in clinical practice. However, an evaluation of the per-protocol set where patients with major protocol violations are excluded is performed additionally and the results are compared with those of the ITT analysis. If a patient discontinues from the study prematurely without having suffered an SSI, missing data for the primary outcome variable will be replaced by using the ICA-r method described by Higgins et al. 2008 [17]. Additionally, sensitivity analyses will be performed by applying alternative methods dealing with missing data such as, e.g., complete case analysis.

### Analysis of secondary variables

The secondary variables will be analyzed descriptively by tabulation of the measures of the empirical distributions. Descriptive p-values of the corresponding statistical tests comparing the intervention groups and associated 95% confidence intervals will be given. For the purpose of hypothesis generation for future trials, subgroup analyses will be performed for patients with different wound status, different severity of wound infection and for patients with and without an applied stoma.

### Adaptive interim analysis

An adaptive interim analysis [18] will be performed after availability of the results for the primary endpoint for a total of 375 randomized patients (i.e., 50% of the number of patients required in a fixed sample size design). The following type I error rates and decision boundaries for the interim and the final analysis are specified:

• overall one-sided type I error rate: 0.025

• boundary for the one-sided p-value of the first stage for accepting the null-hypothesis within the interim analysis: α_0 _= 0.5

• one-sided local type I error rate for testing the null-hypothesis within the interim analysis: α_1 _= 0.0102

• boundary for the product of the one-sided p-values of both stages for the rejection of the null-hypothesis in the final analysis: c_α _= 0.0038

If the trial will be continued with a second stage after the interim analysis (this is possible if for the one-sided p-value p_1 _of the interim analysis p_1_∈]0.0102,0.5[holds true), the results of the interim analysis can be taken into account for a recalculation of the required sample size. If the sample size recalculation leads to the conclusion that more than 1200 patients are required, the study is stopped, because the related treatment group difference is judged to be of minor clinical importance.

Results of the interim analysis will be presented to the Data Safety and Monitoring Board (DSMB) who will advise the Steering Committee of the trial to either terminate or to continue the trial.

### Data Safety Monitoring Board (DSMB)

The DSMB of the trial will consist of three independent members, who will not be involved in the trial. Two are experts with broad experience in surgical RCTs and have served already on DSMB boards and one is a senior biostatistician. The DSMB will assess the Annual Safety Report and will decide whether the trial will be terminated or continued based on the results of the interim analysis. In addition they will assess if one of the termination criteria has been met or if there is any other reason for an early termination of the trial and promptly inform the Steering Committee about their judgment.

### Withdrawals

Patients are free to withdraw trial participation at their own request at any time and without giving reasons for their decision. Moreover, the primary investigator can withdraw study patients, if continuation of the trial would be detrimental to the patient's well being. Withdrawals will be documented in the CRF and in the patient's medical records and all ongoing serious adverse events have to be followed up.

### Trial organization and administration

#### Funding

PROUD was started initially as a single center RCT sponsored by the Department of Surgery, University of Heidelberg, Germany. After a successful application this trial is funded as a multicenter trial by a grant of € 500.000 of Johnson&Johnson Medical Limited, P.O. Box 1988, Kirkton Campus, Livingston EH54, Scotland.

### Ethical considerations

#### Approval

The single center protocol of this trial was approved by the ethics committee of the University of Heidelberg, Germany on March 22, 2010 (Reference number: S-064/2010). After funding was obtained by Johnson&Johnson a substantial amendment was written and approved by the ethics committee of the University of Heidelberg (Date: 29.09.2010, reference number: S-064/2010) and all other ethic committees of the participating centers (between December 8, 2010 and January 11, 2011).

#### Registration

The trial protocol was registered with the German Clinical Trials Register (DRKS; number 00000390) on April 27, 2010. The information was updated after the multicenter protocol was approved by the ethics committee of the University of Heidelberg on October 1, 2010.

#### Good Clinical Practice

The procedures set out in this trial protocol, pertaining to the conduct, evaluation and documentation of this trial, are designed to ensure that all persons involved in the trial abide by Good Clinical Practice and the ethical principles described in the current revision of the Declaration of Helsinki. The trial will be carried out in keeping with local legal and regulatory requirements.

## Discussion

Surgical site infection (SSI) plays a pivotal role for prolonged treatment and further complications, increased health care costs as well as reduced quality of life after open abdominal surgery. They are believed to increase the risk of dying 2-11 fold [19,20], with 77% of these deaths attributed directly to the infection [21].

In the majority of surgical patients SSI was the consequence of almost all interventions until the late 19^th ^century. "Irritative fever" was followed by purulent drainage from the incision and later as sepsis and oftentimes death. When Joseph Lister, in the late 1860s, introduced the principles of antisepsis postoperative infectious morbidity substantially decreased [21]. Ever since there have been substantial and successful efforts to further reduce the number of affected patients and severity of infections with various means: hemostasis, conservation of adequate blood supply, hypothermia prevention, atraumatic tissue handling, and infection control practices such as improved operating room ventilation, sterilisation methods, and the use of antimicrobial prophylaxis [22].

But SSI remain one of the most frequent complications after any type of surgery, ranging as high as 26% depending on types of intervention and definitions of wound infection. This can possibly be attributed in part to the emergence of antibiotic-resistant micro-organisms, larger numbers of elderly surgical patients or those with a variety of chronic and immunocompromising conditions, and greater use of prosthetic implants and organ transplantation [23].

Antimicrobial coated sutures have been amongst the suggestions to further reduce SSI incidence and there is a small number of studies evaluating triclosan coatings in surgery [7,8,11,24]. Still the evaluation of new interventions in surgery remains a major challenge [25]. Given the evidence of two recent single center historically controlled trials with a substantial reduction of more than 50% of wound infections by a triclosan coated suture in abdominal surgery some surgeons might question the demand for further trials. But treatment effectiveness is best evaluated in randomized controlled trials (RCT). The random allocation to one of the treatment groups is the only method to ensure that an observed effect can actually be attributed to the effectiveness of the investigated procedure and not known or unknown extraneous factors. Only RCTs generate data leading to the practice of evidence based medicine on a high level [26]. The Balliol recommendations for evaluating surgery recently have underlined the importance of RCTs and furthermore the manufacturing company has agreed to the funding of such a trial in recognition of the importance of validating the previously mentioned trials in an improved, randomized controlled design [27].

The analysis of the use of triclosan coated sutures in laparotomy previously done by Justinger et al, however big the sample size, has limitations. First of all, in the sequential design that was employed over a period of 2 years, per definition internal validity cannot be assumed with certainty. It is not at all unlikely that over this relatively long period of time other factors in the patients' treatment might have changed and remained unrecorded but may have contributed to the reduction in SSI. Furthermore, with a control of PDS II^® ^sutures in history the intervention group received Vicryl plus^® ^sutures, a material different in structure (monofil versus braided) and resorption (210 versus 70 days). Braided and non braided sutures as well as rapidly absorbable and slowly absorbable ones appear to differ in bacterial adherence [28,29] and interrupted rapid absorbable sutures increase the risk for development of an incisional hernia substantially according to the INLINE systematic review [12].

At first PROUD was initiated as a single center RCT and was transformed into a multicenter RCT once substantial funding was available. Due to this essential amendment the assumptions were adapted to a more conservative approach (reduction of SSI initially assumed to be 73%, now 50%) and an adaptive design given the uncertainties about the true rates of SSI and the difference of SSI between the interventions. The sample size was therefore increased from 200 to 750. This amendment has not affected the interventions, concomitant treatments (antibiotic prophylaxis) of patients and documentation of endpoints. The definition of the primary and secondary endpoints remained unchanged and therefore patients from the single center trial can be analyzed within the multicenter trial. It was helpful to have the experience from the patients treated initially to educate and train the participating centers during the investigator meeting prior to the start of the multicenter recruitment. Practical aspects such as measurements of endpoints or taking and uploading of photograps could be demonstrated to the other centers.

The experience from the INSECT trial and the INLINE systematic review were important for the PROUD trial in two ways. First of all, selection of suture material (slowly absorbable) and suture technique (running suture) is based on the currently best available evidence. INSECT has detected a high rate of wound infections with 16% and PROUD will now show whether a reduction is possible due to the use of antibacterial coating. Secondly, study management was changed as follows: Ethical approval was obtained for all participating center. Afterwards all centers received study related material (sutures, cameras, case report forms) prior to the start of patient recruitment. Finally, during a study meeting all investigators were trained and received the investigator site files. After this meeting each center was able to start screening and randomizing patients immediately. Whether this strategy will help to increase patient recruitment and performance of centers has to be shown in comparison to other RCTs that have been performed by the SDGC.

The PROUD trial with a double blind, parallel group design and independent assessment of wounds by photographic documentation will be adequate to answer the question whether SSI can be reduced by use of antibacterial coated sutures.

## Competing interests

CMS has received reimbursement for lectures given during company organized meetings from Johnson&Johnson and Aesculap. The authors declare that they have no competing interests.

## Authors' contributions

UH, SV, MKD, MK, MWB and CMS planned and designed the PROUD trial. JN, IR, PK, CS and EF have critically reviewed the draft of the protocol. All authors read and approved the final manuscript.

## References

[B1] Bennett-Guerrero E Pappas TN Koltun WA Fleshman JW Lin M Garg J Mark DB Marcet JE Remzi FH George VV Gentamicin-collagen sponge for infection prophylaxis in colorectal surgery N Engl J Med 2010 363 1038 1049 10.1056/NEJMoa1000837 20825316

[B2] Hospital In Europe Link for Infection Control through Surveillance SSI Statistical Report 2004 http://helics.univ-lyon1.fr/documents/HELICS-SSI%20Stat%20Report%202004%20Final%20Version%20180406.pdf

[B3] Seiler CM Bruckner T Diener MK Papyan A Golcher H Seidlmayer C Franck A Kieser M Buchler MW Knaebel HP Interrupted or continuous slowly absorbable sutures for closure of primary elective midline abdominal incisions: a multicenter randomized trial (INSECT: ISRCTN24023541) Ann Surg 2009 249 576 582 10.1097/SLA.0b013e31819ec6c8 19300233

[B4] Zhan C Miller MR Excess length of stay, charges, and mortality attributable to medical injuries during hospitalization JAMA 2003 290 1868 1874 10.1001/jama.290.14.1868 14532315

[B5] Rodeheaver GT Kurtz LD Bellamy WT Smith SL Farris H Edlich RF Biocidal braided sutures Arch Surg 1983 118 322 327 6218796 10.1001/archsurg.1983.01390030054008

[B6] McMurry LM Oethinger M Levy SB Triclosan targets lipid synthesis Nature 1998 394 531 532 10.1038/28970 9707111

[B7] Justinger C Schuld J Sperling J Kollmar O Richter S Schilling MK Triclosan-coated sutures reduce wound infections after hepatobiliary surgery-a prospective non-randomized clinical pathway driven study Langenbecks Arch Surg 2011 10.1007/s00423-011-0786-7 21455702

[B8] Justinger C Moussavian MR Schlueter C Kopp B Kollmar O Schilling MK Antibacterial [corrected] coating of abdominal closure sutures and wound infection Surgery 2009 145 330 334 10.1016/j.surg.2008.11.007 19231586

[B9] Ming X Rothenburger S Nichols MM In vivo and in vitro antibacterial efficacy of PDS plus (polidioxanone with triclosan) suture Surg Infect (Larchmt) 2008 9 451 457 10.1089/sur.2007.061 18687027

[B10] Storch ML Rothenburger SJ Jacinto G Experimental efficacy study of coated VICRYL plus antibacterial suture in guinea pigs challenged with Staphylococcus aureus Surg Infect (Larchmt) 2004 5 281 288 10.1089/sur.2004.5.281 15684799

[B11] Gomez-Alonso A Garcia-Criado FJ Parreno-Manchado FC Garcia-Sanchez JE Garcia-Sanchez E Parreno-Manchado A Zambrano-Cuadrado Y Study of the efficacy of Coated VICRYL Plus Antibacterial suture (coated Polyglactin 910 suture with Triclosan) in two animal models of general surgery J Infect 2007 54 82 88 10.1016/j.jinf.2006.01.008 16487594

[B12] Diener MK Voss S Jensen K Buchler MW Seiler CM Elective midline laparotomy closure: the INLINE systematic review and meta-analysis Ann Surg 2010 251 843 856 10.1097/SLA.0b013e3181d973e4 20395846

[B13] Institute for Medical Informatics SaD, Medical University of Graz, Austria http://www.randomizer.at/

[B14] Paul-Ehrlich Gesellschaft für Chemotherapie e.V Erarbeitung medizinischer Therapieleitlinien Chemotherapie Journal 2010 19

[B15] Bhutani T Jacob SE Triclosan: a potential allergen in suture-line allergic contact dermatitis Dermatol Surg 2009 35 888 889 10.1111/j.1524-4725.2009.01151.x 19389086

[B16] Horan TC Gaynes RP Martone WJ Jarvis WR Emori TG CDC definitions of nosocomial surgical site infections, 1992: a modification of CDC definitions of surgical wound infections Infect Control Hosp Epidemiol 1992 13 606 608 10.1086/646436 1334988

[B17] Higgins JP White IR Wood AM Imputation methods for missing outcome data in meta-analysis of clinical trials Clin Trials 2008 5 225 239 10.1177/1740774508091600 18559412 PMC2602608

[B18] Bauer P Kohne K Evaluation of experiments with adaptive interim analyses Biometrics 1994 50 1029 1041 10.2307/2533441 7786985

[B19] Engemann JJ Carmeli Y Cosgrove SE Fowler VG Bronstein MZ Trivette SL Briggs JP Sexton DJ Kaye KS Adverse clinical and economic outcomes attributable to methicillin resistance among patients with Staphylococcus aureus surgical site infection Clin Infect Dis 2003 36 592 598 10.1086/367653 12594640

[B20] Kirkland KB Briggs JP Trivette SL Wilkinson WE Sexton DJ The impact of surgical-site infections in the 1990s: attributable mortality, excess length of hospitalization, and extra costs Infect Control Hosp Epidemiol 1999 20 725 730 10.1086/501572 10580621

[B21] Mangram AJ Horan TC Pearson ML Silver LC Jarvis WR Guideline for prevention of surgical site infection, 1999. Hospital Infection Control Practices Advisory Committee Infect Control Hosp Epidemiol 1999 20 250 278 quiz 279-280 10.1086/501620 10219875

[B22] Anderson DJ Sexton DJ Kanafani ZA Auten G Kaye KS Severe surgical site infection in community hospitals: epidemiology, key procedures, and the changing prevalence of methicillin-resistant Staphylococcus aureus Infect Control Hosp Epidemiol 2007 28 1047 1053 10.1086/520731 17932825

[B23] Hedrick TL Heckman JA Smith RL Sawyer RG Friel CM Foley EF Efficacy of protocol implementation on incidence of wound infection in colorectal operations J Am Coll Surg 2007 205 432 438 10.1016/j.jamcollsurg.2007.04.042 17765159

[B24] Fleck T Moidl R Blacky A Fleck M Wolner E Grabenwoger M Wisser W Triclosan-coated sutures for the reduction of sternal wound infections: economic considerations Ann Thorac Surg 2007 84 232 236 10.1016/j.athoracsur.2007.03.045 17588420

[B25] Diener MK Simon T Buchler MW Seiler CM Surgical evaluation and knowledge transfer-methods of clinical research in surgery Langenbecks Arch Surg 2011 10.1007/s00423-011-0775-x 21424797

[B26] McLeod RS Issues in surgical randomized controlled trials World J Surg 1999 23 1210 1214 10.1007/s002689900649 10552108

[B27] Ergina PL Cook JA Blazeby JM Boutron I Clavien PA Reeves BC Seiler CM Altman DG Aronson JK Barkun JS Challenges in evaluating surgical innovation Lancet 2009 374 1097 1104 10.1016/S0140-6736(09)61086-2 19782875 PMC2855679

[B28] Rodeheaver GT Beltran KA Green CW Faulkner BC Stiles BM Stanimir GW Traeland H Fried GM Brown HC Edlich RF Biomechanical and clinical performance of a new synthetic monofilament absorbable suture J Long Term Eff Med Implants 1996 6 181 198 10167360

[B29] Chu CC Williams DF Effects of physical configuration and chemical structure of suture materials on bacterial adhesion. A possible link to wound infection Am J Surg 1984 147 197 204 10.1016/0002-9610(84)90088-6 6364858

